# Recent development of mitochondrial metabolism and dysfunction in osteoarthritis

**DOI:** 10.3389/fphar.2025.1538662

**Published:** 2025-02-13

**Authors:** Pengchao Guo, Ahmad Alhaskawi, Safwat Adel Abdo Moqbel, Zhijun Pan

**Affiliations:** ^1^ Emergency Department, The Second Affiliated Hospital of Zhejiang University School of Medicine, Hangzhou, China; ^2^ Department of Orthopedics, The First Affiliated Hospital of Zhejiang University School of Medicine, Hangzhou, China; ^3^ Department of Orthopedics, The Second Affiliated Hospital of Zhejiang University School of Medicine, Hangzhou, China

**Keywords:** osteoarthritis, cartilage degradation, mitochondrial dysfunction, mitochondrial metabolism, inflammation

## Abstract

Osteoarthritis is a degenerative joint disorder characterized by cartilage degradation, synovial inflammation, and altered subchondral bone structure. Recent insights have identified mitochondrial dysfunction as a pivotal factor in OA pathogenesis, contributing to chondrocyte apoptosis, oxidative stress, and extracellular matrix degradation. Disruptions in mitochondrial dynamics, including impaired biogenesis, mitophagy, and metabolic shifts from oxidative phosphorylation to glycolysis, exacerbate cartilage damage by promoting the production of reactive oxygen species and matrix-degrading enzymes such as ADAMTS and MMPs. This review explores the molecular mechanisms underlying mitochondrial dysfunction in OA, emphasizing its role in cartilage homeostasis and inflammation. Furthermore, it highlights emerging therapeutic strategies targeting mitochondrial pathways, including antioxidants, mitophagy enhancers, and metabolic modulators, as potential interventions to mitigate disease progression, which offer promising avenues for advancing personalized and disease-modifying treatments in OA.

## Introduction

**FIGURE 1:**
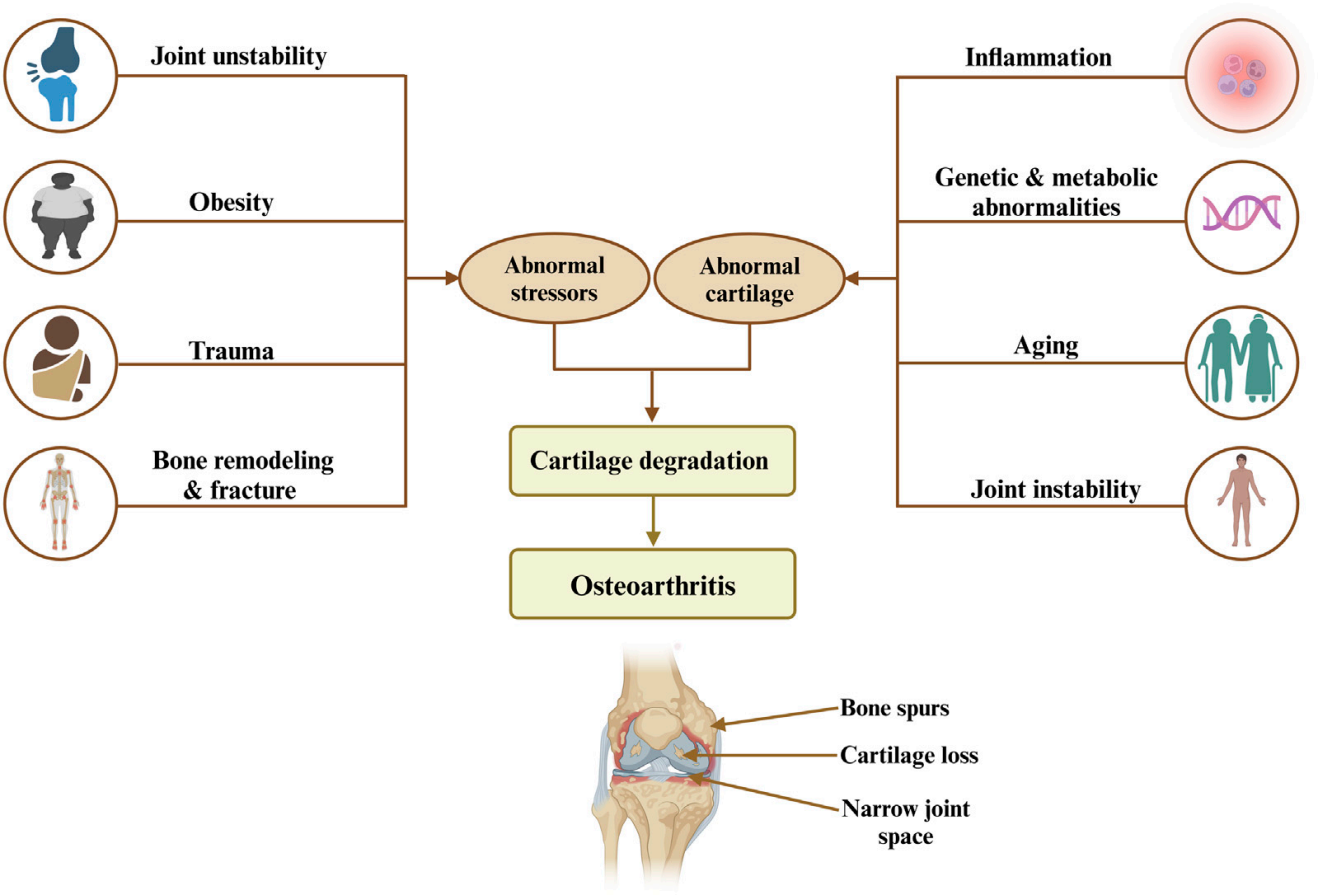
Risk factors and mechanisms leading to osteoarthritis highlight the interplay between abnormal stress, cartilage degradation, and resulting joint damage, including bone spurs, cartilage loss, and narrow joint space.

Osteoarthritis (OA) is a chronic joint disorder recognized as the most common form of arthritis, affecting millions worldwide and leading to significant pain, disability, and a reduced quality of life (Barnett, 2018; Glyn-Jones et al., 2015). This progressive disease is primarily marked by the degeneration of articular cartilage, which cushions the ends of bones in joints, alongside changes in subchondral bone structure, synovial inflammation, and the formation of bony growths, or osteophytes. Although OA has the potential to affect any joint, it predominantly affects weight-bearing joints such as the hips, hands, knees, and spine (Glyn-Jones et al., 2015). Several key risk factors, including genetics, age, gender, joint injury, obesity, and repetitive stress influence OA (Figure 1). Age remains the most significant factor, as cartilage naturally wears down over time. Genetic predispositions increase susceptibility, particularly in hand and knee OA, while women, particularly post-menopause, have a higher prevalence of OA, which has been hypothesized to relate to differences in hormonal balance and associated comorbidities (Zhang and Zhou, 2024; Loeser et al., 2016). Furthermore, obesity accelerates OA in weight-bearing joints by increasing mechanical load and contributing inflammatory mediators. In addition, joint injuries and occupations involving repetitive motions further stress joints, leading to cartilage breakdown. Along with that, metabolic disorders and low bone density also play a role, linking OA with systemic inflammation and joint degeneration (Sampath et al., 2023; Datta et al., 2017; Masson and Krawetz, 2020). Currently, there is no cure for OA, and the available treatments mainly focus on relieving pain, improving joint function, and enhancing quality of life (Yao et al., 2023). The primary goal of OA management is to control symptoms and maintain mobility, rather than halting disease progression. As such, OA remains an urgent medical condition requiring further investigation to uncover effective disease-modifying treatments (Table 1) (Mihalko et al., 2023; Maqbool et al., 2021).

**TABLE 1:** A summary of osteoarthritis treatment modalities.

Treatment category	Treatment	Description	Effectiveness	Benefits and limitations	References
Non-Pharmacologic	Physical Therapy	A structured exercise regimen improves joint flexibility and muscle strength and reduces pain	Effective for mild-to-moderate OA	Well-tolerated and adaptable; however, adherence can be challenging for long-term results	Kolasinski et al. (2019), Skou and Roos (2019)
	Weight Management	Reducing body weight to alleviate stress on load-bearing joints	Effective, especially for knee OA	Significant pain reduction and improved function; requires sustained lifestyle changes	Messier et al. (2005), Lim et al. (2022)
	Aquatic Exercise	Low-impact exercise in water that minimizes joint stress while building strength	Effective for symptom relief and joint flexibility	Beneficial for those with mobility limitations; the availability of facilities can be a barrier	Bartels et al. (2016), French et al. (2022)
Pharmacologic	NSAIDs (naproxen, ibuprofen)	Anti-inflammatory drugs that reduce pain and inflammation	Moderate pain relief	Effective for pain but can cause cardiovascular and gastrointestinal side effects with long-term use	Bannuru et al. (2015), Magni et al. (2021)
	Acetaminophen	Analgesic primarily for mild pain management	Modest effectiveness	Safer option but less effective than NSAIDs for OA pain; liver toxicity risk with high doses	Machado et al. (2015), Bannwarth (2006)
	Duloxetine	Antidepressant with pain-relieving properties used off-label for OA.	Moderate relief for chronic pain	May benefit those with coexisting depression; limited by side effects, including nausea and fatigue	Wang et al. (2017), Osani and Bannuru (2019)
	Topical NSAIDs (e.g., diclofenac gel)	Topical application of anti-inflammatory drugs to reduce local joint pain	Effective for mild-to-moderate pain relief	Fewer systemic side effects than oral NSAIDs; limited penetration for deeper joints	Derry et al. (2016), Bariguian Revel et al. (2020)
Intra-Articular Injections	Corticosteroids	Anti-inflammatory injections directly into the joint for acute pain management	Short-term relief; benefits decrease over time	Effective for flare-ups but can accelerate joint degeneration with repeated use	McAlindon et al. (2014), Ayhan et al. (2014)
	Hyaluronic Acid	Injected to improve joint lubrication and reduce stiffness	Mixed effectiveness	Short-term improvement in function and pain relief; costly and limited to mild OA cases	Bannuru et al. (2015), Magni et al. (2021), Migliore and Procopio (2015)
Emerging Therapies	Platelet-rich plasma (PRP)	Injection of concentrated platelets derived from the patient’s blood to stimulate joint repair and promote tissue regeneration	Variable results; promising in early OA stages	Potential regenerative benefits; limited evidence, and not standardized	Dai et al. (2017), Gato-Calvo et al. (2019), Moretti et al. (2022)
	Stem Cell Therapy	Injection of mesenchymal stem cells to stimulate cartilage regeneration	Early evidence is promising but unproven	Shows potential in slowing cartilage loss; expensive and experimental	Vangsness et al. (2014), Thoene et al. (2023)
	Gene Therapy	Targeted gene modification to alter cellular processes causing cartilage degeneration	Experimental; early studies show potential	Could target underlying causes of OA; high technical and ethical challenges	Madry and Cucchiarini (2016), Li et al. (2023), Zhang et al. (2020)
Surgical	Arthroscopy	Minimally invasive joint surgery to remove damaged tissue	Limited long-term benefit for OA	Suitable only for specific cases, often has minimal effect on OA progression	McGrory et al. (2016), Felson (2010)
	Osteotomy	Surgical realignment of bones to relieve joint stress, typically around the knee	Effective for younger patients with early OA	Helps preserve natural joints, delaying need for joint replacement; requires significant recovery time	Brouwer et al. (2014), Peng et al. (2021)
	Joint Replacement	Replacement of the joint with a prosthesis, typically for severe OA.	Highly effective for advanced OA	Major surgery with long recovery; most effective for pain relief and function in end-stage OA.	Shan et al. (2014), Günther et al. (2021)
Supplements and Alternative	Glucosamine and Chondroitin	Supplements aimed at supporting cartilage health	Inconsistent evidence for effectiveness	Widely used but benefits remain debated; generally safe with few side effects	Zhu et al. (2018), McAlindon et al. (2000), Conrozier and Lohse (2022)
	Curcumin (from Turmeric)	Natural anti-inflammatory supplement used to relieve joint pain	Some mild pain relief	Few side effects; efficacy not consistent across studies, though safe for adjunctive use	Daily et al. (2016), Perkins et al. (2017), Chin (2016)
	Cannabidiol (CBD)	Non-psychoactive component of cannabis with anti-inflammatory properties	Preliminary evidence for pain relief	Limited studies; generally well-tolerated, though legal restrictions affect availability	Wong et al. (2020), Frane et al. (2022)

Mitochondria are essential organelles in joint tissues, playing a critical role in cellular energy production, metabolism, and overall homeostasis. Their primary function is to generate adenosine triphosphate (ATP) through oxidative phosphorylation, providing energy necessary for a variety of cellular processes. This energy supports cellular activities such as protein synthesis, ion transport, and cell division, which are crucial for the maintenance and function of joint tissues, including chondrocytes in cartilage and synoviocytes in the synovial lining (Giacomello et al., 2020; Chakrabarty and Chandel, 2022). Mitochondria also play a crucial role in cellular signaling pathways within joint tissues. They are involved in modulating the activity of enzymes that control matrix remodeling, a process that ensures the continual turnover and renewal of cartilage. The mitochondria, through their regulation of cellular metabolism, influence the synthesis of molecules involved in maintaining the ECM, which is essential for joint integrity, as it ensures cartilage retains its elasticity and load-bearing properties. Furthermore, mitochondria contribute to the regulation of redox balance in chondrocytes, a critical factor in cellular stress responses and overall tissue health (Tait and Green, 2012; Pickles et al., 2018; Stoolman et al., 2022). The maintenance of mitochondrial biogenesis is another key aspect of their role in joint tissue function. This process ensures that the quantity and function of mitochondria are sufficient to meet the metabolic demands of chondrocytes, particularly during periods of increased cellular activity, such as in response to mechanical load or metabolic stress. The regulation of mitochondrial biogenesis and turnover is essential for maintaining the energy balance of joint tissues and ensuring the proper function of chondrocytes over time (Pickles et al., 2018; Liu et al., 2023a). Structurally, mitochondria possess a unique double-membrane system, with the inner membrane extensively folded into cristae to increase surface area, optimizing ATP production (Giacomello et al., 2020; Chakrabarty and Chandel, 2022). Research on mitochondrial function and dysfunction has expanded significantly, especially due to its implications for human health. Mitochondrial dysfunction has been linked to numerous diseases, encompassing neurodegenerative disorders, metabolic syndromes, and aging. As a result, understanding mitochondrial biology offers promising avenues for therapeutic interventions. Many studies have increasingly investigated the role of elevated mitochondrial ROS in accelerating cartilage degeneration and OA development (He Y. et al., 2020; Kan et al., 2021). Studies indicate that excessive ROS production in chondrocytes, driven by mitochondrial dysfunction, leads to cellular damage and oxidative stress, promoting inflammation and apoptosis within joint tissues. Research conducted by Hu et al. revealed that the use of natural antioxidants (Quercetin), mitigates ROS levels in OA by reducing chondrocyte apoptosis and inflammation, thereby slowing cartilage breakdown (Hu et al., 2019). Similarly, Chen et al. investigated the pathway in temporomandibular joint OA, finding that mitochondrial ROS-induced oxidative stress activates the HIF-1α/TFRC pathway, leading to ferroptosis and further cartilage degeneration (Chen et al., 2024). Furthermore, mitochondrial dysfunction leads to reduced ATP production, thereby impairing chondrocyte function and undermining cartilage maintenance (Zhang Y. et al., 2022; Yi et al., 2021). Animal experiments have strongly supported the association between mitochondrial dysfunction and OA progression. In OA mouse models, disruption of mitochondrial respiration has been shown to accelerate both synovitis and cartilage degradation, emphasizing the essential role of mitochondrial health in maintaining joint integrity (Ansari et al., 2020a; Yi et al., 2022). Additionally, mitochondrial DNA (mtDNA) mutations are particularly problematic, as they interfere with the electron transport chain, reducing ATP production while simultaneously increasing the generation of ROS (Kopinski et al., 2021). Shen et al. highlight how dysfunctions in mitochondrial dynamics, especially in the regulatory proteins involved in fission and fusion, can lead to an accumulation of damaged mitochondria, further exacerbating inflammation and tissue breakdown in OA joints (Chen Q. et al., 2023). Furthermore, mitochondrial biogenesis has been identified as a promising therapeutic approach for OA, with emerging evidence indicating its potential to alleviate pain and restore joint functionality. In the study by Gao et al., the compound dimethyl fumarate (DMF) was used to enhance mitochondrial biogenesis in OA rat model. DMF activated the nuclear factor erythroid 2-related factor 2 (Nrf2) pathway, which plays a vital role in enhancing antioxidant defenses and maintaining mitochondrial health. The activation led to an increase in mitochondrial biogenesis markers, including TFAM, NRF1, and PGC-1α, which improved mitochondrial function and reduced oxidative stress within chondrocytes. The enhanced mitochondrial function through Nrf2-mediated biogenesis not only restored energy balance in the cells but also alleviated pain behaviors in OA rats, highlighting mitochondrial biogenesis as a valuable target in managing OA (Gao et al., 2022).

In general, mitochondrial dysfunction and metabolic alterations are key contributors to the onset and progression of OA. Thus, our article offers an in-depth analysis of the mechanisms connecting mitochondrial dysfunction to OA pathophysiology, highlighting the potential for developing targeted therapies aimed at modulating mitochondrial metabolic pathways to slow disease progression and enhance patient outcomes.

## Determinants of mitochondrial dysfunction in osteoarthritis pathogenesis

**FIGURE 2:**
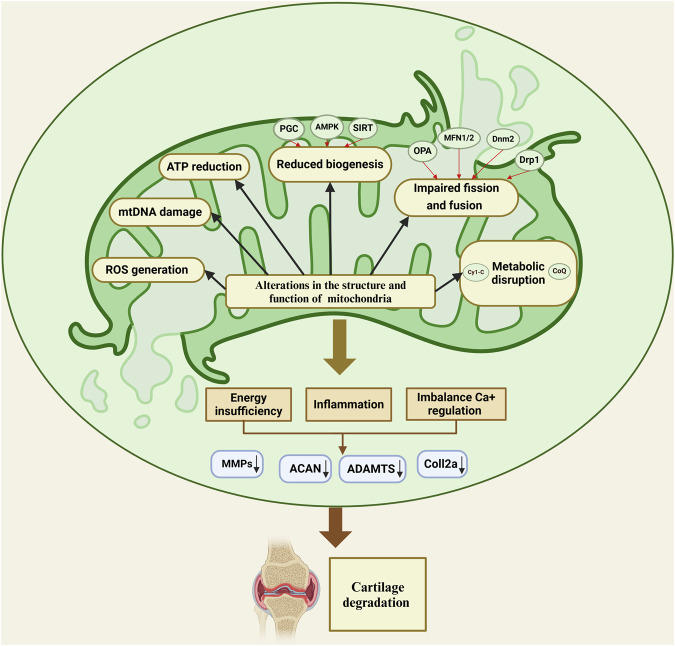
Mitochondrial dysfunction in osteoarthritis disrupts energy production, calcium balance, and inflammation, leading to cartilage degradation through impaired biogenesis, fission-fusion imbalance, and metabolic disruption.

Mitochondrial dysfunction is a critical underlying factor in the initiation and progression of OA. Evidence shows that these mitochondrial irregularities emerge before cartilage degradation begins, contributing significantly to the death of chondrocytes (Figure 2) (Zhang et al., 2024; Cheung et al., 2024). Mitochondrial dysfunction in OA arises from multiple factors, with oxidative stress serving as a primary contributor. It not only damages mitochondrial DNA (mtDNA) but also compromises respiratory function and initiates mitochondria-driven pathways of cell death. ROS and inflammatory cytokines produced by synovial cells and chondrocytes intensify oxidative stress, furthering the damage to mitochondria (Ansari et al., 2020b; Arra et al., 2020). Specifically, cytokines such as TNF-α and IL-1β are recognized to impair mitochondrial respiration, diminish ATP production, and reduce mitochondrial activity in chondrocytes, thereby exacerbating mitochondrial dysfunction in OA (Doll et al., 2015; López-Armada et al., 2006). Moreover, maintaining appropriate mitochondrial dynamics involves balanced fission and fusion processes, which is essential for ensuring optimal mitochondrial functionality. A reduced fusion alongside increased fission leads to fragmented, dysfunctional mitochondria, which diminishes ATP production and heightens ROS generation (Tilokani et al., 2018; Adebayo et al., 2021). Zhang et al. reported that moderate mechanical stress promoted the upregulation of mitochondrial fusion proteins OPA1, MFN1, and MFN2, in chondrocytes stimulated with IL-1β. These proteins have a crucial role in maintaining mitochondrial integrity by promoting mitochondrial fusion, which helps counteract excessive fission and fragmentation induced by inflammatory stress. The upregulating MFN1/2 and OPA1, moderate stress supports mitochondrial health, reduces apoptosis, and preserves chondrocyte function. In contrast, excessive mechanical stress suppresses the expression of these mitochondrial fusion proteins, impairing mitochondrial dynamics and resulting in heightened cellular dysfunction and apoptosis (Zhang J. et al., 2021). Alterations in processes like mitochondrial mitophagy and biogenesis are also recognized as contributing factors to mitochondrial dysfunction in OA. Also, a study investigated the effects of Urolithin A (UA) on OA and found that UA stimulates mitophagy via the PINK1-Parkin pathway, which is essential for removing damaged mitochondria in OA. Therefore, by promoting mitophagy, UA improved mitochondrial function, reduced cartilage degradation, and alleviated pain in OA models (D'Amico et al., 2022). Another study, by Deng et al. found that PARP12 inhibits PINK1/Parkin-dependent mitophagy, PARP12 interacts with ISG15 to increase ISGylation of mitochondrial fusion proteins MFN1/2, which, in turn, reduces their ubiquitylation and SUMOylation, ultimately inhibiting mitophagy (Deng et al., 2024). In addition, Functionalized Prussian blue nanoparticles (RAPA@MPB-MMP9 NPs) have been found to promote mitophagy in synovial macrophages, stabilize mitochondrial health, and reduce inflammation (Qi et al., 2024). Sun et al. demonstrated that overexpressing Sestrin2 (Sesn2) in the spinal cord of a rat model alleviated osteoarthritis-related pain by enhancing mitochondrial biogenesis and reducing neuroinflammation. This method activated AMPK/PGC-1α signaling, which restored mitochondrial function, decreased inflammatory cytokines, and suppressed glial activation, highlighting the management effect of Sesn2 in OA pain (Sun et al., 2022). Furthermore, Yu et al. showed that mitochondrial transplantation from bone marrow mesenchymal stem cells (BMSCs) significantly enhanced mitochondrial biogenesis in osteoarthritic chondrocytes, which reduced oxidative stress, restored mitochondrial function, and elevated ATP production (Yu et al., 2022). Vega-Letter et al. investigated the therapeutic potential of mitochondria transplantation derived from umbilical cord mesenchymal stromal cells (Mito-MSC) as an innovative cell-free therapy for OA demonstrating that Mito-MSC were effectively internalized by chondrocytes, synovial macrophages, and fibroblasts, leading to enhanced mitochondrial functionality and increased ATP production while transcriptomic analysis revealed the activation of stress response and antiviral immune pathways, indicating protective mechanisms against cartilage degeneration in a collagenase-induced OA murine model (Vega-Letter et al., 2025). In addition, the interplay between mitochondria and the endoplasmic reticulum (ER) is essential for maintaining cellular homeostasis, with disruptions in this crosstalk contributing to the pathogenesis of OA (Zhao and Sheng, 2024). One critical aspect of this interaction is the regulation of calcium signaling between the mitochondria and the ER. Mitochondria-associated membranes (MAMs), where calcium transfer from the ER to mitochondria plays a pivotal role in regulating mitochondrial bioenergetics, cell survival, and apoptosis (Zhao and Sheng, 2024; Rodríguez-Arribas et al., 2017; Morciano et al., 2018). In OA, dysregulated calcium signaling, resulting from mitochondrial dysfunction or ER stress, leads to disrupted calcium homeostasis, exacerbating mitochondrial dysfunction and promoting chondrocyte apoptosis (Delco and Bonassar, 2021). Furthermore, excessive calcium accumulation within mitochondria can trigger the mitochondrial permeability transition pore (MPTP) opening, resulting in the release of pro-apoptotic factors such as cytochrome c, which in turn activates downstream apoptotic pathways and accelerates cartilage degradation (Rizzuto et al., 2012; Zhou et al., 2022). The impairment of mitochondrial-ER communication, particularly through aberrant calcium flux, contributes to oxidative stress and exacerbates mitochondrial fragmentation, which further disrupts cellular integrity and accelerates OA progression (Morcillo et al., 2024). Understanding these factors offers valuable insights into potential therapeutic methods, paving the way for treatments that target mitochondrial health to mitigate disease progression and improve joint function.

## Mitochondrial morphological and functional alterations in osteoarthritis

Mitochondrial morphological alterations and changes in mitochondrial number are critical features of OA. In OA-affected chondrocytes, mitochondria often display increased fragmentation, disrupted cristae, and irregular shapes, all of which compromise their functional integrity. Additionally, a reduction in mitochondrial number is frequently observed, limiting the cell’s capacity for energy production and contributing to cartilage degeneration (Guidotti et al., 2015). A study found that mitochondrial fragmentation in OA chondrocytes is regulated by the TBK1-mediated phosphorylation of DRP1 at Ser637, which prevents excessive mitochondrial fission and supports mitophagy, ultimately helping to the preservation of mitochondrial quality (Hu et al., 2023). Chen et al. identified the AMPK-SIRT3 positive feedback loop as a crucial regulator of OA progression by enhancing mitochondrial quality in chondrocytes, supporting mitophagy, and reducing oxidative damage, thus mitigating OA-related cellular degeneration (Chen et al., 2021). Beyond morphological alterations, mitochondrial function is compromised in OA, as evidenced by reduced ATP production and mitochondrial respiration. A study found that REDD1 deficiency exacerbates OA severity by impairing mitochondrial biogenesis and autophagy in articular cartilage. Specifically, REDD1 deficiency reduces the expression of autophagy-related proteins, including ATG5 and LC3 and mitochondrial biogenesis markers, leading to decreased mitochondrial DNA content, lower ATP levels, and heightened chondrocyte apoptosis under stress (Alvarez-Garcia et al., 2017). Wang et al. concluded that mitochondrial biogenesis in osteoarthritic chondrocytes is impaired due to diminished SIRT1 and AMPK activity, which decreases PGC-1α expression, and demonstrated that activating AMPK pharmacologically can restore mitochondrial function and counteract pro-catabolic responses, offering a therapeutic avenue for OA (Wang et al., 2015). OA chondrocytes undergo a profound shift in mitochondrial metabolism, transitioning from oxidative phosphorylation to glycolysis, by elevated extracellular acidification rates, increased lactate production, and diminished mitochondrial respiration. This metabolic reprogramming disrupts ATP synthesis and elevates oxidative stress, ultimately compromising mitochondrial integrity and function, indicating mitochondrial metabolism’s pivotal role in OA pathogenesis (Zheng et al., 2021). Reprogramming mitochondrial metabolism in M1 macrophages through camouflaged meta-Defensome nanoparticles effectively induces a phenotypic shift from the pro-inflammatory M1 state to the anti-inflammatory M2 state. This metabolic reprogramming reduces ROS and NO production, restores aerobic respiration, and increases TFAM expression, which collectively mitigates synovial inflammation and slows osteoarthritis progression (Zhang L. et al., 2022). Similarly, mitochondrial dysfunction in OA was targeted by using the NAHA-CaP/siCA9 nanocarrier to address metabolic imbalances in synovial macrophages, reducing nitric oxide (NO) levels and thereby mitigating inflammation and cartilage degradation associated with mitochondrial impairment (Yan et al., 2023).

## The role of mitochondrial dysfunction in maintaining cartilage integrity and chondrocyte health

Many studies demonstrated that mitochondrial dysfunction in chondrocytes plays an important role in affecting cartilage matrix production, along with promoting chondrocyte senescence and apoptosis, collectively contributing to the initiation and progression of OA. Chondrocytes are essential for maintaining the extracellular matrix (ECM) of cartilage, which is vital for its structural integrity and function. These specialized cells synthesize and regulate key ECM components, such as proteoglycans and collagen, ensuring that the cartilage remains resilient under mechanical stress (Welting et al., 2018). Chondrocytes adapt their metabolic activity to the cartilage’s microenvironment, adjusting ECM turnover as needed to preserve cartilage health and resist degradation processes that can lead to OA (Alexopoulos et al., 2003). Dysfunctional mitochondrial activity can lead to heightened oxidative stress, interfere with ATP synthesis, and cause a buildup of damaged lipids and proteins, collectively promoting chondrocyte apoptosis (Blanco et al., 2011). Chondrocyte apoptosis plays a significant role in cartilage degeneration by diminishing the cell population responsible for ECM synthesis, ultimately compromising cartilage integrity. This apoptotic process disrupts the balance of cartilage homeostasis, as chondrocytes are essential for maintaining matrix turnover and structural resilience. For instance, Thomas et al. revealed that elevated chondrocyte apoptosis is closely linked to both the onset and severity of cartilage degradation, highlighting its contribution to OA progression (Thomas et al., 2011). Mitochondrial dysfunction extends its impact beyond chondrocyte apoptosis, significantly affecting the ECM’s composition and structural organization within cartilage tissue. Mitochondrial impairment in OA increases ROS levels, which induces oxidative stress and promotes chondrocyte death, while enzymes like MMP3 and ADAMTS-5 actively degrade cartilage (Xiao et al., 2024). Furthermore, a study shows that Icariin (ICA) mitigates these effects by enhancing mitochondrial stability, reducing ROS, and inhibiting matrix-degrading enzymes, ultimately preserving cartilage integrity and decelerating OA progression (Xiao et al., 2024). Nevertheless, elevated AURKA levels in OA promote mitochondrial dysfunction by degrading SOD2, leading to increased ROS and accelerated cartilage degradation through enhanced expression of matrix-degrading enzymes (Yang et al., 2019). Thus, silencing AURKA has been shown to counteract these effects, suggesting it as a promising target for maintaining mitochondrial function and cartilage integrity in OA (Yang et al., 2019). Mitochondrial dysfunction disrupts the production and organization of proteoglycans and collagen in cartilage, weakening its structural integrity. This impairment reduces collagen stability and alters proteoglycan arrangement, making cartilage more prone to degradation. Guidotti et al. investigated the glycogen synthase kinase-3β (GSK3β) role in OA chondrocytes and found that GSK3β inhibition leads to significant ECM degradation and chondrocyte hypertrophy (Guidotti et al., 2017), in addition, GSK3β inhibition disrupts mitochondrial function, increases oxidative stress, and promotes matrix metalloproteinase activity (Guidotti et al., 2017). Another study also found that mitochondrial dysfunction in OA chondrocytes led to increased ROS production, reduced collagen type II synthesis, and elevated activity of MMPs, particularly MMP-13, resulting in significant ECM degradation (Liu H. et al., 2019).

## Mitochondrial metabolic activity and inflammatory processes in osteoarthritis

Inflammation is increasingly recognized as a central mechanism in the pathogenesis of OA, driving joint deterioration and cartilage degradation. Distinct from the acute, high-grade inflammation characteristic of rheumatoid arthritis, OA is typified by chronic, low-grade inflammation predominantly driven by the innate immune system (Robinson et al., 2016; Siebuhr et al., 2016). This inflammatory response is initiated by biomechanical stress and cellular damage within the joint environment, triggering the release of pro-inflammatory cytokines including, IL-1β, IL-6, and TNF-α. They further accelerate cartilage breakdown through the induction of matrix-degrading enzymes, perpetuating tissue degradation and joint damage (Robinson et al., 2016; Siebuhr et al., 2016). In addition, various factors beyond cytokines, such as damage-associated molecular patterns (DAMPs), chemokines, and adipokines. DAMPs, which are released from damaged cartilage and subchondral bone, interact with pattern recognition receptors like toll-like receptors (TLRs) on synovial cells and chondrocytes. This interaction activates inflammatory signaling pathways, including NF-κB, thereby intensifying local inflammation and contributing to the progression of joint degradation (Gratal et al., 2022; Lambert et al., 2020). Along with that, chemokines such as CCL2, CCL5, and CXCL8 are small signaling proteins that recruit immune cells to the site of inflammation and elevated in OA-affected joints, attracting macrophages and neutrophils that release inflammatory cytokines, further exacerbating tissue damage (Raymuev, 2018). Moreover, adipokines, including leptin, resistin, and adiponectin, are cytokine-like molecules secreted by adipose tissue and are found at higher levels in OA, particularly in obese individuals. These adipokines not only promote cartilage breakdown by inducing MMPs but also increase pro-inflammatory cytokines production, linking metabolic dysregulation to enhanced joint inflammation (Table 2) (Xie and Chen, 2019). Synovitis plays a particularly significant role, not only contributing to joint pain and stiffness but also facilitating cartilage and bone degradation through interactions among immune cells in the synovium (Sanchez-Lopez et al., 2022), this inflammatory milieu disrupts the equilibrium between catabolic and anabolic processes in cartilage, creating a vicious cycle of joint degeneration and osteophyte formation (Robinson et al., 2016; Berenbaum and van den Berg, 2015). Multiple studies indicate that modulating mitochondrial metabolism could be a viable therapeutic method for OA, offering the potential to mitigate both inflammatory responses and mitochondrial dysfunction. Jin et al. presented curcumin’s anti-inflammatory effects and its role in addressing mitochondrial dysfunction in OA. Curcumin reduces the synthesis of pro-inflammatory cytokines, thereby alleviating inflammation in cartilage tissue. In addition, curcumin promotes mitophagy via the AMPK/PINK1/Parkin pathway, which helps maintain mitochondrial health (Jin et al., 2022). Another study investigates the therapeutic potential of koumine in treating OA, and the results showed that koumine decreased the levels of inflammatory markers (IL-6, IL-1β, TNF-α) and ECM-degrading enzymes (ADAMTS5, MMP13) in both chondrocytes and rat models of OA, furthermore, it enhances mitochondrial function by stabilizing mitochondrial membrane potential and reducing ROS generation (Kong et al., 2024). Farnaghi et al. showed that mitochondria-targeted antioxidants play a protective role in OA by reducing mitochondrial oxidative stress, which in turn lowers inflammation and prevents cartilage degradation (Farnaghi et al., 2017). Focusing on the mitochondrial pyruvate carrier (MPC) holds therapeutic promise in OA by modulating mitochondrial metabolism and reducing oxidative stress. The MPC facilitates the transport of pyruvate into mitochondria, fueling oxidative phosphorylation and ATP production, which are essential for chondrocyte function and cartilage maintenance. Dysfunction in the MPC pathway is associated with elevated oxidative stress and an imbalance in mitochondrial metabolism, which promotes inflammation and cartilage degeneration in OA. Modulating MPC activity may help restore mitochondrial function and decrease ROS production, thereby reducing inflammation, enhancing chondrocyte survival, and potentially slowing the progression of OA (Fang et al., 2016; Coleman et al., 2018; Ghosh et al., 2016). A study by Zhang et al. focuses on the contributions of hypoxia-inducible factors HIF-1α and HIF-2α in OA development. HIF-1α is shown to support cartilage homeostasis by promoting chondrocyte survival, ECM synthesis, and adaptation to hypoxia, thus exerting protective effects. In contrast, HIF-2α acts as a catabolic factor, enhancing matrix-degrading enzymes and pro-inflammatory mediators expression, which contribute to cartilage degradation (Zhang F. J. et al., 2015; Zeng et al., 2022). Furthermore, blocking HOTAIR, was showed a significant reduction in oxidative stress, decreases in inflammatory cytokines, and preservation of ECM components. Additionally, HOTAIR is shown to regulate OA pathogenesis through its interaction with ADAM10 and miR-222-3p, where silencing HOTAIR or modulating the miR-222-3p/ADAM10 pathway may mitigate chondrocyte injury and ECM degradation (Wang J. et al., 2021). Moreover, the long non-coding RNA NEAT1 promotes inflammation and ECM degradation in OA by negatively regulating miR-193a-3p, which in turn targets SOX5 (Liu et al., 2020). Qiao et al. investigates the therapeutic potential of Asperosaponin VI (ASA VI), which activates Sirtuin 3 (Sirt3). Utilizing an *in vitro* model of IL-1β-induced chondrocytes and an *in vivo* OA model, ASA VI was shown to enhance chondrocyte viability, reduce apoptosis, and suppress inflammatory markers, including IL-6 and TNF-α (Qiao et al., 2024). Nevertheless, Hong et al. suggested Glaucocalyxin A (GLA) as a potential therapeutic for OA, showing that GLA reduces inflammation and protects cartilage by inhibiting MAPK and NF-κB signaling pathways. In IL-1β-stimulated chondrocytes and a DMM mouse model, GLA suppressed inflammatory markers (iNOS, COX-2), decreased MMP13 levels, and preserved ECM components like collagen type II and SOX9. These effects collectively slowed cartilage degradation and reduced OA severity (Hong et al., 2024).

**TABLE 2:** The roles of cytokines, chemokines, and adipokines in osteoarthritis pathogenesis.

Molecule	Role in OA pathogenesis	References
IL-1β	Promotes cartilage degradation by upregulating MMPs and ADAMTS; inhibits collagen synthesis	Goldring and Otero (2011), Kapoor et al. (2011)
TNF-α	Induces inflammation and cartilage matrix degradation through MMPs; enhances apoptosis in chondrocytes	Goldring and Otero (2011), Molnar et al. (2021)
IL-6	Stimulates inflammatory responses and osteoclast-mediated bone resorption; contributes to synovitis	Narazaki et al. (2017), Hunter and Bierma-Zeinstra (2019)
IL-15	Regulates immune cell activity in synovium and perpetuates chronic inflammation	Molnar et al. (2021), Warner et al. (2020)
IL-17	Promotes cartilage destruction and osteophyte formation; synergizes with IL-1β and TNF-α in inflammation	Xiao et al. (2023), Amatya et al. (2017)
IL-18	Induces nitric oxide production, MMP expression, and chondrocyte apoptosis; enhances cartilage breakdown	Szilagyi et al. (2023), Matsui et al. (2003)
IL-8	Drives neutrophil infiltration in joints, aggravating synovitis and promoting catabolic activity in cartilage	Chauffier et al. (2012), Takahashi et al. (2015)
LIF	Modulates cartilage matrix turnover and inflammatory response in OA synovium	Hui et al. (1998), Jiang et al. (2014)
CCL2 (MCP-1)	Attracts monocytes/macrophages to synovium, exacerbating inflammation and tissue damage	Molnar et al. (2021), Raghu et al. (2017)
CCL5 (RANTES)	Involved in recruitment of immune cells to the joint; promotes synovial inflammation and cartilage degradation	Molnar et al. (2021), Raghu et al. (2017)
Leptin	Promotes chondrocyte hypertrophy and MMP production; contributes to cartilage catabolism and inflammation	Cordero-Barreal et al. (2021), Bao et al. (2010)
Resistin	Induces pro-inflammatory cytokine expression; contributes to synovitis and cartilage breakdown	Senolt et al. (2007), Fadda et al. (2013)
Adiponectin	Dual role: promotes MMP expression and inflammation, but may also enhance cartilage repair under certain conditions	Ouchi and Walsh (2007), Ruscica et al. (2012)

**FIGURE 3:**
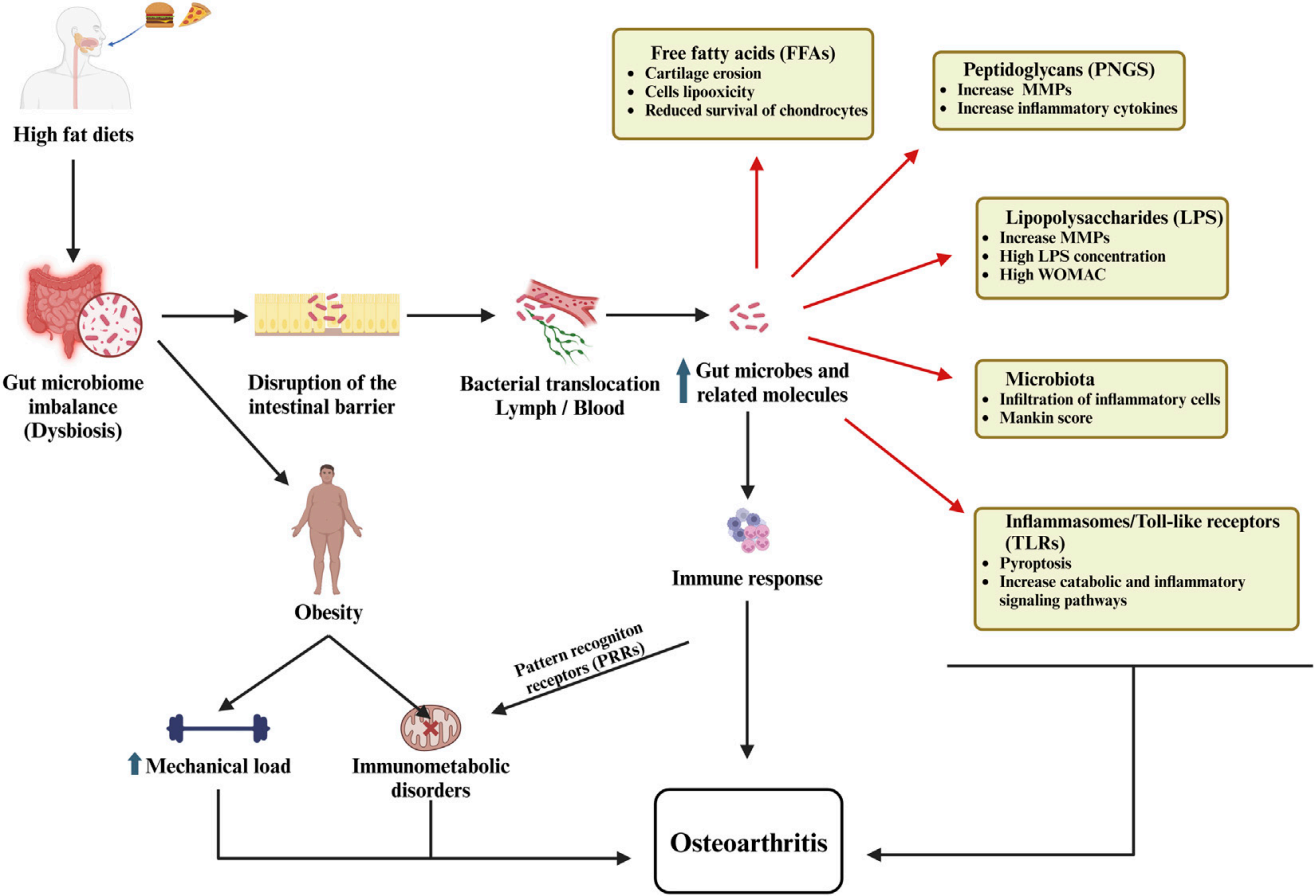
The role of the gut microbiome in osteoarthritis illustrates how dysbiosis, intestinal barrier dysfunction, bacterial translocation, immune activation, and metabolic disturbances collectively contribute to the development and progression of the disease.

Recent research highlights the significant influence of gut microbiota on regulating mitochondrial metabolism and controlling inflammatory pathways in OA. Gut microbiota produces metabolites that influence immune responses and mitochondrial function in chondrocytes, thereby affecting inflammation and cartilage integrity in OA (Lian et al., 2022). Dysbiosis, or an imbalance in gut microbial composition, has been linked to increased systemic inflammation and altered mitochondrial metabolism, particularly in obesity-associated OA. This microbial imbalance promotes low-grade inflammation and oxidative stress, contributing to degradation of the cartilage and disease progression (Liu Y. et al., 2019; Jiménez-Muro et al., 2023). Key microbial components, such as lipopolysaccharides (LPS), initiate inflammatory pathways that compromise mitochondrial function in joint tissues, leading to metabolic imbalances and mitochondrial dysfunction within chondrocytes (Figure 3). This cascade perpetuates a pro-inflammatory and oxidative environment, intensifying cartilage breakdown and OA advancement (Collins et al., 2015). Thus, the use of probiotic treatment in OA offers potential by rebalancing gut microbiota, reducing inflammation, and supporting mitochondrial stability (Jiménez-Muro et al., 2023).

## Role of mitochondrial metabolism and apoptosis in osteoarthritis

Mitochondria serve as essential regulators of apoptosis in response to cellular stress, its dysfunction leads to activation of apoptotic pathways, which are intrinsic and extrinsic. In OA chondrocytes the mitochondrial dysfunction is marked by oxidative stress, impaired electron transport chain activity, and loss of mitochondrial membrane potential, which can drive ECM degradation and chondrocyte apoptosis through the activation of caspases (Hwang and Kim, 2015). A study investigates how lysosomal dysfunction contributes to an imbalance in apoptosis regulation in OA by promoting chondrocyte death. It shows that impaired lysosomal function causes mitochondrial damage and the pro-apoptotic BAX pathway activation, which facilitates Cytochrome c release from mitochondria and triggers apoptosis (Ansari et al., 2020a). In another study, the release of Cytochrome c from mitochondria was shown to be a critical step in NO-induced apoptosis in osteoarthritic chondrocytes, this release activates downstream apoptotic pathways, including caspase-9 and caspase-3, ultimately resulting in cellular death (He B. et al., 2020). The matrix degradation and apoptosis in OA were mitigated by S-Equol through activation of the PI3K/Akt signaling pathway, which reduced oxidative stress and downregulated matrix-degrading enzymes MMPs, therefore, S-Equol preserved mitochondrial function, decreased NO and ROS levels, and inhibited pro-apoptotic protein expression (Huang et al., 2021). Furthermore, SIRT3, a mitochondrial protein, has been shown to mitigate chondrocyte damage under high-glucose conditions by promoting autophagy and reducing apoptosis, indicating that supporting mitochondrial function could enhance autophagic repair pathways in OA cartilage (Wang et al., 2024). Furthermore, the protein BNIP3L has been recognized as a critical regulator of mitophagy in OA models, where its dysregulation exacerbates mitochondrial damage and limits autophagic responses, thereby promoting cartilage degradation (Yang et al., 2024). Huang et al. examine the impact of a thermosensitive hydrogel delivering a leptin inhibitor (LI) on cartilage repair in OA, targeting the STAT3/REDD1/mTORC1 signaling pathway. By inhibiting this pathway, the LI effectively reduces leptin-induced suppression of autophagy in OA chondrocytes. Specifically, the inhibition of STAT3 activation leads to upregulation of REDD1, which in turn suppresses mTORC1 activity, thereby restoring autophagic flux, reducing chondrocyte apoptosis, and promoting cellular survival (Huang et al., 2023). Nevertheless, orphan nuclear receptor subfamily four group A member 1 (NR4A1) upregulation in OA chondrocytes is linked to mitochondrial dysfunction, as it promotes mitochondrial fragmentation and activates apoptotic pathways; the receptor enhances the translocation of dynamin-related protein 1 (Drp1) to the mitochondria, facilitating mitochondrial fission, which disrupts mitochondrial integrity and triggers apoptosis (Liu et al., 2022). Xing et al. investigated the effect of mitochondrial dynamics markers. They presented the disruptions in mitochondrial dynamics particularly the TOM20 protein involved in mitochondrial protein import, which lead to mitochondrial dysfunction and contributes to cellular stress and chondrocyte apoptosis in OA. Additionally, imbalances in other markers like MFN1 and MFN2, which regulate mitochondrial fusion, result in excessive mitochondrial fragmentation, exacerbating oxidative stress, inflammation, and matrix degradation (Xing et al., 2023) (Table 3). Investigating mitochondria-targeted therapies was the focus of many studies aiming to prevent chondrocyte apoptosis in OA. Among these, mitochondria-targeted antioxidants have shown particular promise by directly addressing oxidative damage within chondrocytes. Unlike conventional antioxidants, which may have limited effectiveness within the mitochondrial environment, these targeted compounds specifically accumulate in the mitochondria, reducing ROS at their primary source. By neutralizing ROS, mitochondrial antioxidants such as mitoquinone (MitoQ) and SkQ1 protect mitochondrial DNA, proteins, and lipids from oxidative damage, thereby preserving mitochondrial integrity and function (Farnaghi et al., 2017; Kim et al., 2010; Huang et al., 2024). This protection helps to stabilize mitochondrial membrane potential, maintain ATP production, and ultimately prevent the release of pro-apoptotic factors that trigger chondrocyte death. A study identified that mitochondrial-targeted therapy with the peptide SS-31 effectively preserves chondrocyte viability and mitigates cartilage degeneration in models of posttraumatic osteoarthritis (PTOA). By selectively binding to cardiolipin on the mitochondrial membrane, SS-31 stabilizes mitochondrial cristae structure, reduces ROS production, and prevents mitochondrial-induced apoptosis (Delco et al., 2018). Liu et al. demonstrated that α-ketoglutarate (α-KG) alleviates OA by restoring mitochondrial function and reducing oxidative stress through the activation of the PINK1-Parkin mitophagy pathway. Their study found that α-KG supplementation in OA models promotes chondrocyte proliferation, inhibits apoptosis, and regulates ECM homeostasis, thus mitigating cartilage degeneration. Notably, the inhibiting mitophagy with Mdivi-1 reversed these benefits, underscoring mitophagy’s critical role in α-KG’s therapeutic effects on OA (Liu et al., 2023b). Moreover, Wang et al. demonstrates that Tanshinone I reduces chondrocyte apoptosis in OA by the inhibition of NF-κB, which drives inflammation-induced cell death. In both *in vivo* models and IL-1β-stimulated chondrocytes, Tanshinone I significantly decreased apoptosis, preserved chondrocyte viability, and maintained cartilage integrity (Wang X. et al., 2019). In addition, Cyclosporin A plays a role in reducing chondrocyte apoptosis by inhibiting the opening of the mitochondrial permeability transition pore, thereby preventing the release of pro-apoptotic factors, therefore, reduce oxidative stress, helps to stabilize mitochondrial integrity, and protect chondrocytes from apoptosis (Verhagen et al., 2015). Targeting Bcl-2 family proteins is another method utilized by mitochondria-specific small molecules to prevent apoptosis. Pro-apoptotic proteins such as Bak and Bax mediate mitochondrial outer membrane permeabilization (MOMP), a key event in apoptosis initiation (Dewson and Kluck, 2009; Dadsena et al., 2021). Small molecules that inhibit Bak and Bax, or upregulate anti-apoptotic Bcl-2 proteins, effectively block the mitochondrial apoptotic pathway. Compounds like ABT-199 and Bcl-2 mimetics enhance chondrocyte survival by preventing MOMP, maintaining mitochondrial integrity, and reducing apoptosis in OA and related degenerative diseases (Velentza et al., 2023; Montell et al., 2015; Zhang X. et al., 2015). Beyond this, Bao et al. demonstrate that circFAM160A2 suppresses OA chondrocytes apoptosis by sponging miR-505-3p, leading to increased SIRT3 expression, which in turn stabilizes mitochondria and reduces oxidative stress (Bao et al., 2021). Another study showed that circATRNL1 expression is significantly reduced in IL-1β-treated chondrocytes, a model for OA, and the overexpression of circATRNL1 reduces ECM degradation and chondrocyte apoptosis by sponging miR-153-3p (Wang K. F. et al., 2021). Pharmacological activation of AMPK boosts SIRT3 levels, which in turn reduces oxidative stress, stabilizes mitochondrial integrity, and restores key mitochondrial functions such as ATP production and respiration. By deacetylating mitochondrial proteins like SOD2 and OGG1, SIRT3 enhances antioxidant capacity and supports DNA repair (Chen et al., 2018). Overall, addressing mitochondrial dysfunction and apoptosis offers a compelling therapeutic approach for osteoarthritis by directly targeting the critical pathways involved in cartilage degeneration.

**TABLE 3:** Primary regulators of mitochondrial biogenesis and dynamics in osteoarthritis (OA).

Regulators	Expression level	Functional role	References
AMPK	Downregulated	Increase levels of MMP-3, MMP-13, and nitric oxide	Huang et al. (2021), Petursson et al. (2013)
PGC-1a	Downregulated	Reduce oxidative stress and suppress the pro-catabolic response, thereby preventing the advancement of cartilage degradation in OA	Sun et al. (2022), Zhao et al. (2014)
TFAM	Downregulated	Suppress mitochondrial biogenesis while promoting catabolic reactions to IL-1β, leading to an increased release of MMP-3, MMP-13, and nitric oxide	Wang et al. (2015), Bao et al. (2021)
SIRT1	Downregulated	Drive the progression of OA by elevating apoptotic markers, MMP-13, type X collagen, acetylated NF-κB p65, and ADAMTS-5 levels	Wang et al. (2024), Matsuzaki et al. (2014)
MFN1	Downregulated	Promote mitochondrial fission activity in chondrocytes under inflammatory conditions	Shin et al. (2019), Wang et al. (2020)
MFN2	Upregulated	Worsen inflammation and OA progression, downregulate Parkin expression, and induce metabolic alterations	Xing et al. (2023), Xu et al. (2020)
Drp1	Upregulated	Promote mitochondrial fission activity in chondrocytes under inflammatory conditions	Liu et al. (2022), Wang et al. (2020)

## Mitochondrial metabolism and its role in cartilage matrix degradation in osteoarthritis

Cartilage matrix degradation is a defining characteristic of OA, with mitochondrial dysfunction serving as a key contributor to this pathological process. The impact of mitochondrial dysfunction on ECM degradation in cartilage is mediated through multiple interconnected pathways. Among these, the upregulation of MMPs, particularly MMP-13, plays a pivotal role. MMP-13 facilitates the breakdown of collagen and other ECM components, thereby promoting pathological ECM degradation and tissue remodeling in OA (Cui et al., 2017). Jing et al. showed that iron overload contributes to mitochondrial damage and oxidative stress, leading to excessive ROS production. This cascade destabilizes mitochondrial membrane potential, initiates apoptosis, and upregulates MMPs, particularly MMP-3 and MMP-13, which degrade the ECM, in addition, the calcium chelator BAPTA-AM can mitigate these effects by reducing iron influx, thereby protecting mitochondrial integrity, lowering ROS levels, and decreasing MMP expression, which decreases the degradation of cartilage matrix (Jing et al., 2021). Many studies indicated that mitochondrial dysfunction causes oxidative stress, subsequently activating pathways such as MAPK and NF-κB, which further stimulate the expression of MMP, as well as ADAMTS-4 and ADAMTS-5, which target cartilage collagen and aggrecan, respectively (Loeser, 2009; Kunkel et al., 2016; Xu et al., 2024). Furthermore, the inhibition of mitochondrial respiratory chain (MRC) complexes, specifically complexes III and V, leads to increased expression and release of MMP-1 and MMP-3, enzymes involved in ECM breakdown, while reducing MMP-13 expression, which is crucial in early OA, this dysregulation results in an imbalance that favors cartilage degradation and reduces proteoglycan levels (Cillero-Pastor et al., 2013). Chen et al. evaluates the chondroprotective effects of ferulic acid (FA) on hydrogen peroxide-stimulated chondrocytes and found that FA significantly protects the cartilage matrix from breakdown, through decreasing the oxidative stress markers, the expression of pro-inflammatory cytokines, and MMP-1 (Chen et al., 2010). In addition, Song et al. demonstrate that hydrogen sulfide (H₂S) prevents cartilage matrix degradation in OA by reducing oxidative stress and inhibiting inflammatory pathways, which in turn downregulates MMPs, thus preserving cartilage integrity (Song et al., 2024). Ni et al. found that cathepsin B (CTSB) leaks from lysosomes into the cytoplasm, where it induces mitochondrial dysfunction by disrupting mitochondrial membrane integrityand increasing ROS production, therefore increasing the cartilage matrix damage (Ni et al., 2022). Another study by Zhang et al. demonstrates that miR-140-5p reduces cartilage matrix damage in OA by targeting and inhibiting CTSB, which disrupts the CTSB/NLRP3 inflammasome pathway, thereby decreasing chondrocyte pyroptosis and inflammatory cytokine release (Zhang L. et al., 2021). These findings highlight the critical role of mitochondrial dysfunction in regulating matrix-degrading enzymes within chondrocytes, a process that significantly contributes to cartilage matrix degradation in OA.

## Autophagy and mitochondrial metabolism in osteoarthritis

Autophagy, a vital cellular mechanism responsible for removing damaged organelles and maintaining energy balance, is also dysregulated in OA, resulting in the accumulation of dysfunctional mitochondria and increased susceptibility to chondrocyte apoptosis. The interaction between mitochondrial dysfunction and impaired autophagy creates a vicious cycle that accelerates extracellular matrix degradation, inflammation, and joint degeneration (Cheng et al., 2023; Matsuzaki et al., 2018; Dalmao-Fernández et al., 2024). Kim et al. explores the mitochondrial dysfunction in OA which causes excessive ROS production, impaired mitochondrial biogenesis, and chondrocyte apoptosis, contributing to cartilage degradation, and dysfunction activates mitophagy pathways, such as BNIP3-mediated autophagy, which exacerbates ECM breakdown (Kim et al., 2021). Xue et al. highlights the significance of inhibiting the PI3K/AKT/mTOR signaling pathway to enhance autophagy in articular chondrocytes. They found that inflammatory stimuli, such as IL-1β, suppress chondrocyte autophagy, leading to reduced expression of proteins (LC3, Beclin1) and autophagy-related genes (Atg5, Atg7), contributing to cartilage degradation. The inhibition of the PI3K/AKT/mTOR pathway reverses these effects, increasing autophagy, enhancing chondrocyte survival, and reducing inflammation (Xue et al., 2017). Another study explored the protective role of oxymatrine (OMT) in mitigating IL-1β-induced chondrocyte damage, emphasizing its ability to activate autophagy through the suppression of the AKT/mTOR signaling pathway. OMT enhances autophagy by reducing p62 levels and increasing LC3-II expression, both key markers of autophagic activity. The activation of autophagy mitigates chondrocyte apoptosis, reduces oxidative stress, and protects against cartilage matrix degradation associated with IL-1β-induced damage (Lu et al., 2024). Further enhancement of the autophagy process and targeting mitochondrial dysfunction provide a significant strategic approach to developing effective OA therapies. Wang et al. demonstrated that metformin mitigates IL-1β-induced mitochondrial dysfunction in chondrocytes by activating the SIRT3/PINK1/Parkin-dependent mitophagy pathway. This process reduces ROS, improves mitochondrial function, and restores cartilage ECM balance by enhancing collagen II level and decreasing the expression of MMP3 and MMP13 (Wang C. et al., 2019). Zhao et al. study highlights the PINK1/Parkin-mediated mitophagy pathway as a critical mechanism for maintaining mitochondrial health, and rapamycin treatment was shown to enhance mitophagy, reduce ROS, and restore mitochondrial membrane potential (Zhao et al., 2024). In addition, zinc restores mitochondrial function by promoting the PINK1/Parkin pathway, increasing key autophagy markers such as LC3-II and Beclin1, which facilitate the clearance of damaged mitochondria (Huang et al., 2020). Furthermore, inhibiting p66shc reduces ROS levels and improves mitochondrial function, enhancing autophagic activity. This increase in autophagy facilitates the removal of damaged mitochondria, preserves chondrocyte viability, and helps maintain cartilage integrity (Shin et al., 2020). As a result, mitochondrial-targeted therapies offer the potential for enhancing chondrocyte survival in OA and modulating autophagy.

## Future direction

Mitochondrial dysfunction serves as a critical factor in the progression of OA, influencing chondrocyte apoptosis, oxidative stress, and ECM degradation. Addressing these challenges, future research should focus on innovative strategies targeting mitochondrial pathways, leveraging advancements in mitochondrial therapeutics, genetic modulation, and novel drug delivery systems. Developing mitochondria-targeted therapeutics, which include antioxidants, mitophagy modulators, and mitochondrial biogenesis activators, offers immense potential for mitigating mitochondrial dysfunction in OA. Many studies have investigated a range of treatments that may demonstrate efficacy as mitochondria-targeted therapeutics. Small molecules like MitoQ and SkQ1 have shown promise in preclinical studies by preserving mitochondrial membrane potential and reducing mitochondrial ROS, which are critical for maintaining chondrocyte viability and cartilage integrity (Farnaghi et al., 2017), as SS-31, which stabilize mitochondrial membranes and enhance ATP production, represent another avenue for therapeutic intervention. Expanding these findings through clinical trials is important to evaluate their efficacy and long-term safety in OA management. Huang et al. revealed that metformin protects against cartilage damage and mitigates mitochondrial dysfunction in OA models by leveraging its AMP-independent AMPK activation pathway. The study highlighted that metformin inhibits v-ATPase through its binding to the lysosomal PEN2-ATP6AP1 axis, thereby activating AMPK without disrupting cellular energy ratios. This activation mitigates mitochondrial dysfunction and oxidative stress. In animal models, metformin’s modulation of the AMPK pathway reduced markers of cartilage degradation and inflammation, demonstrating its potential to preserve cartilage integrity (Ma et al., 2022). Furthermore, Na et al. investigate the therapeutic potential of Coenzyme Q10 (CoQ10) encapsulated in micelles for OA. Using a rat model of monosodium iodoacetate-induced OA, demonstrates that CoQ10-micelles significantly alleviate OA symptoms, including tissue destruction, pain, and inflammation. The treatment reduced inflammatory cytokines and catabolic markers while inhibiting necroptosis-associated cell death pathways involving RIP1, RIP3, and pMLKL. Additionally, CoQ10-micelles exhibited superior chondroprotective effects compared to standard CoQ10, preserving cartilage integrity and reducing bone erosion (Na et al., 2022).

Genetic and epigenetic strategies offer novel insights into the regulation of mitochondrial function in OA. Key regulators, such as SIRT3 and PGC-1α, influence mitochondrial biogenesis, oxidative phosphorylation, and ROS detoxification (Deng et al., 2019). Employing advanced tools like CRISPR gene-editing technology could enable precise modulation of these pathways, restoring mitochondrial function and energy homeostasis in chondrocytes. A study explores CRISPR/Cas9 gene editing targeting NGF, IL-1β, and MMP13. While NGF ablation alleviates pain but worsens joint damage, IL-1β and MMP13 deletion reduce cartilage degradation and inflammation. Multiplex editing balances pain relief and structural preservation, highlighting CRISPR/Cas9’s potential for precise OA therapies (Zhao et al., 2019). Additionally, exploring the role of non-coding RNAs, including miRNAs and lncRNAs, could reveal further therapeutic targets related to mitochondrial metabolism in OA. Moreover, identifying mitochondrial biomarkers is crucial for early diagnosis and monitoring of OA progression. Biomarkers indicative of mitochondrial dysfunction, such as changes in mtDNA integrity or mitochondrial dynamics proteins (e.g., DRP1, MFN2), could aid in stratifying patients for personalized treatment approaches. Incorporating such biomarkers into clinical workflows may also improve the assessment of therapeutic efficacy for mitochondrial-targeted interventions (Court et al., 2024; Lin et al., 2024). Furthermore, combining mitochondria-targeted therapies with other treatments, such as antioxidants, anti-inflammatory agents, or cartilage repair strategies, could provide a comprehensive approach to managing OA (Tian et al., 2024). For example, integrating autophagy modulators with mitochondrial antioxidants could enhance the clearance of dysfunctional mitochondria while mitigating oxidative stress, synergistically preserving chondrocyte health and cartilage function (Zheng et al., 2023). Advanced drug delivery systems, such as nanoparticles and liposomal carriers, could enhance the bioavailability and specificity of mitochondrial-targeted therapies. For instance, the development of mitochondrial-targeting nano-prodrugs has shown promise in delivering therapeutic agents directly to mitochondria *in vitro*, improving efficacy in reducing ROS and apoptosis in OA models (Huang et al., 2024). Sustained-rel tailored for synovial tissue environments could further optimize therapeutic outcomes while minimizing systemic side effects. On the other hand, robust animal models remain indispensable for validating mitochondrial-targeted strategies and bridging preclinical findings to clinical applications (Galuzzi et al., 2018). However, despite their potential, toxicity concerns must be thoroughly examined. The toxicity associated with these therapies often stems from their effects on mitochondrial function, off-target impacts, and potential disruption of cellular homeostasis. Excessive modulation of mitochondrial biogenesis or mitophagy can disrupt the balance of ROS, leading to oxidative stress and apoptosis in both target and non-target tissues (Chen W. et al., 2023; Bhatti et al., 2017). Although reports on mitochondrial therapy toxicity specifically in OA are limited, findings from related diseases and preclinical studies offer valuable insights. Mitochondrial uncouplers, such as 2,4-dinitrophenol (DNP), which are being investigated for metabolic reprogramming, have been linked to hepatotoxicity and hyperthermia due to their non-specific disruption of mitochondrial function (Goedeke and Shulman, 2021; Shrestha et al., 2021). Excessive use of mitochondrial antioxidants may interfere with normal ROS signaling, potentially impairing immune responses and reducing cellular adaptability (Craige et al., 2024; Kowalczyk et al., 2021; Jomova et al., 2023). Future research should prioritize optimizing therapeutic protocols and conducting translational studies to assess the long-term efficacy and safety of these interventions on joint health and OA progression. By addressing these critical areas, future research holds the potential to revolutionize the treatment landscape for OA, offering disease-modifying therapies that restore mitochondrial function, preserve cartilage integrity, and improve patient outcomes.

## Conclusion

Mitochondrial dysfunction stands as a central pathogenic driver in the development and progression of OA, profoundly influencing the integrity of articular cartilage. Through mechanisms including diminished mitochondrial respiration, heightened oxidative stress, and impaired ATP production, mitochondrial dysfunction accelerates chondrocyte apoptosis and promotes the expression of matrix-degrading enzymes such as MMPs and aggrecanases. These processes significantly contribute to the breakdown of the ECM, which is a hallmark of OA pathophysiology. Furthermore, the disruption of mitochondrial dynamics, including an imbalance in mitochondrial fission and fusion, impedes mitochondrial quality control, leading to the accumulation of dysfunctional mitochondria that further perpetuate cellular stress and exacerbate cartilage degradation. In addition to the well-characterized role of oxidative stress in mitochondrial dysfunction, emerging evidence underscores the importance of metabolic reprogramming in OA. Chondrocytes in OA are increasingly driven by glycolytic pathways, shifting away from oxidative phosphorylation. This metabolic shift impairs ATP production and exacerbates the production of ROS, creating a vicious cycle that promotes chronic inflammation and matrix degradation. Furthermore, the perturbation of mitochondrial autophagy (mitophagy) and biogenesis plays a critical role in the progression of OA, as defective mitophagy exacerbates mitochondrial damage, while impaired mitochondrial biogenesis hinders the capacity of cells to maintain mitochondrial homeostasis. Recent advancements in mitochondrial-focused therapeutic strategies, such as the use of mitochondria-targeted antioxidants like MitoQ and SkQ1, hold significant promise in mitigating oxidative stress within OA-affected tissues. These compounds, by directly targeting the mitochondria, offer a refined approach to neutralizing ROS at their primary source, thus preserving mitochondrial function and reducing chondrocyte apoptosis. In parallel, interventions aimed at enhancing mitophagy through pharmacological agents or gene therapies represent a novel strategy to restore mitochondrial integrity by promoting the selective removal of damaged mitochondria. Additionally, activation of mitochondrial biogenesis, as seen with compounds like dimethyl fumarate (DMF), presents a promising avenue to restore mitochondrial function and alleviate the metabolic disruptions inherent in OA. As our understanding of the molecular underpinnings of mitochondrial dysfunction in OA deepens, future research should aim to refine these therapeutic approaches and develop strategies that more effectively restore mitochondrial health. This includes exploring novel mitochondrial-targeted drug delivery systems, such as nanoparticles, to enhance the specificity and bioavailability of therapeutic agents. Furthermore, the identification of mitochondrial biomarkers will be critical for early diagnosis, monitoring disease progression, and evaluating the efficacy of therapeutic interventions.
